# Molecular imaging of orthotopic prostate cancer with nanobubble ultrasound contrast agents targeted to PSMA

**DOI:** 10.1038/s41598-021-84072-5

**Published:** 2021-02-25

**Authors:** Yu Wang, Al Christopher De Leon, Reshani Perera, Eric Abenojar, Ramamurthy Gopalakrishnan, James P. Basilion, Xinning Wang, Agata A. Exner

**Affiliations:** 1 Department of Radiology, Case Western Reserve University, 10900 Euclid Avenue, BRB 330, Cleveland, OH 44106 USA; 2 Department of Ultrasound, Peking University People’s Hospital, Beijing, 100044 China; 3 Department of Biomedical Engineering, Case Western Reserve University, 11100 Euclid Ave, Wearn Building B49, Cleveland, OH 44106 USA

**Keywords:** Cancer imaging, Urological cancer

## Abstract

Ultrasound imaging is routinely used to guide prostate biopsies, yet delineation of tumors within the prostate gland is extremely challenging, even with microbubble (MB) contrast. A more effective ultrasound protocol is needed that can effectively localize malignancies for targeted biopsy or aid in patient selection and treatment planning for organ-sparing focal therapy. This study focused on evaluating the application of a novel nanobubble ultrasound contrast agent targeted to the prostate specific membrane antigen (PSMA-targeted NBs) in ultrasound imaging of prostate cancer (PCa) in vivo using a clinically relevant orthotopic tumor model in nude mice. Our results demonstrated that PSMA-targeted NBs had increased extravasation and retention in PSMA-expressing orthotopic mouse tumors. These processes are reflected in significantly different time intensity curve (TIC) and several kinetic parameters for targeted versus non-targeted NBs or LUMASON MBs. These, may in turn, lead to improved image-based detection and diagnosis of PCa in the future.

## Introduction

In the United States, prostate cancer (PCa) is the most commonly diagnosed cancer and the second most-deadly cancer for males. It is thus critical to find a reliable and minimally invasive tool for PCa diagnosis. The incidence of PCa increased in the last decade of the twentieth century due to the introduction of prostate-specific antigen (PSA) blood tests and a dramatic increase in PSA screening, as more and more patients were diagnosed with low-risk or indolent disease^1^. It has been previously reported that ∼70% of men with elevated PSA do not have PCa^2,3^, so the high false positive rates from PSA blood tests continue to be a serious problem^4^ contributing to potential overdiagnosis and overtreatment. Unlike liver or lung tumors, CT scans are less sensitive to PCa^5^. Currently, magnetic resonance imaging (MRI) is considered the best modality for PCa diagnosis because it is the most sensitive to soft tissue changes^6^. However, due to the bulky equipment, high cost, and limited adaptability, this technology still faces some challenges for widespread application in this area. Globally, the availability, accessibility and cost factors of MRI present an even greater obstacle to broad adoption. Therefore, the diagnosis of PCa still predominantly relies on systematic random biopsies^7^ despite a high incidence of complications associated with the biopsy procedure^8^, and a significant need for repeated biopsies to identify suspected malignancies^9^. This necessitates the need for an imaging modality that can effectively localize prostate malignancies for targeted biopsy^10^ or assist in patient selection and treatment planning for organ-sparing focal therapy.

PCa may be visible on standard B-mode transrectal ultrasound (TRUS). However, the performance reported in the literature varies widely with sensitivities ranging from 8 to 88% and specificities ranging from 42.5 to 99%^7^. Therefore, B-mode ultrasound is widely considered to be an insufficiently accurate method for tumor detection^11^. In order to improve the accuracy of TRUS in the diagnosis of PCa, contrast-enhanced ultrasound (CEUS) has been proposed. It is important to note that CEUS is like enhanced CT or MRI because it requires the injection of a contrast agent to indicate the blood flow distribution of different tissues and organs. However, compared to CT scans, radiation exposure is significantly reduced. In addition, costs are lower than MRI^5^. Ultrasound also could reduce patient waiting time as it does not require the use of complex equipment and is therefore easy to implement. These advantages support the use of CEUS as the most effective method for detecting and monitoring changes in the prostate^6^.

Clinically, intravascular ultrasound contrast agents (UCA) – e.g. microbubbles (MBs)—have been used to observe changes in vascularity, and contrast-specific imaging modes are even able to image microcirculation at a capillary scale^12^. Those are important, because angiogenesis has been shown to be critical for the development of prostate tumors from small indolent lesions below 2 mm to clinically significant diseases^13^. Despite the reported improvements in tumor detection rate^14^, targeted biopsies based on visual interpretation of CEUS alone are not considered viable to replace systematic biopsy^10^. Ultrasound MBs currently used for CEUS are limited by their size (1–10 μm), which can only produce blood pool contrast. These bubbles are also rapidly cleared from the cancer tissue, resulting in short-acting contrast agents. Also, while MBs targeted to the vascular endothelium of tumors have been examined clinically^15^, MBs cannot be targeted to specific tumor cell antigens because they are unable to efficiently pass through the submucosal layers into tumor tissue^16^. To overcome these challenges, a new kind of UCA is needed^17^.

Lipid-stabilized nanobubbles (NBs) with a particle size of less than 1000 nm for use as ultrasound contrast agents have been a research focus in recent years. They can overcome the limitations of clinically used MBs that they remain in the blood vessels, and therefore have the potential for targeted US imaging and treatment of tumors^18^. In view of the above, our team has recently developed an ultra-stable NB contrast agent (100–500 nm in diameter) composed of a perfluoropropane gas core stabilized by a phospholipid, propylene glycol (PG) and glycerol shell. This combination of materials has resulted in nanobubbles with unique physicochemical properties^19^ including strong echogenicity at clinical frequencies using standard nonlinear imaging sequences and persistence in circulation over tenfold longer than clinical MBs. Previous studies have also showed that these NBs can extravasate beyond the vasculature and are retained for a substantial time within the tumor parenchyma^20^. Moreover, equipping NBs with specific antibodies or ligands may be a simple means of producing specific targeted delivery systems^21^. Prostate specific membrane antigen (PSMA) is a type II integral membrane protein, which has high levels of specific expression in both androgen-dependent PCa and androgen-independent PCa^22^. Currently, a variety of ligands against PSMA have been extensively applied in fluorescence imaging, MRI, and molecular nuclear medicine of PCa^23–25^. We have recently shown the capabilities of PSMA-targeted NBs in targeting PCa in vitro and in flank tumors^20^.

**Figure 1:**
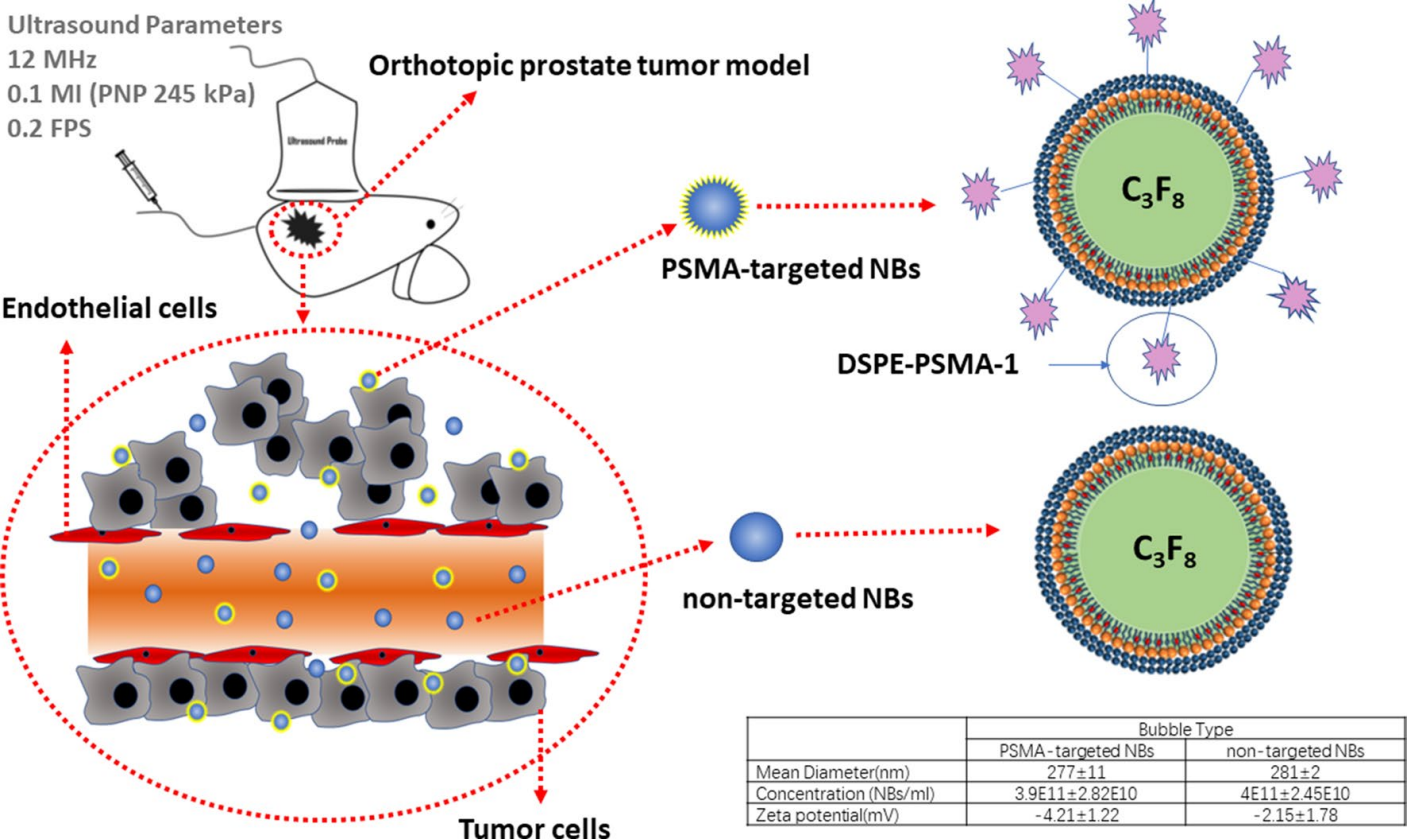
The schematic diagram of tumor model and PSMA-targeted NBs and non-targeted NBs.

Our aim in the current study was to investigate PSMA-targeted NBs for US imaging of PCa in vivo using a more clinically relevant orthotopic prostate tumor model in nude mice (Fig. 1). Given the robust nature of the NB-enhanced ultrasound, we also used the technique to examine the effect of PSMA-targeting efficiency on tumor progression and size in the same model. This may provide methods for relevant studies on targeted ultrasound NBs.

## Results

### Contrast-enhanced ultrasound imaging of orthotopic prostate tumors using PSMA-targeted NBs and LUMASON

**Figure 2:**
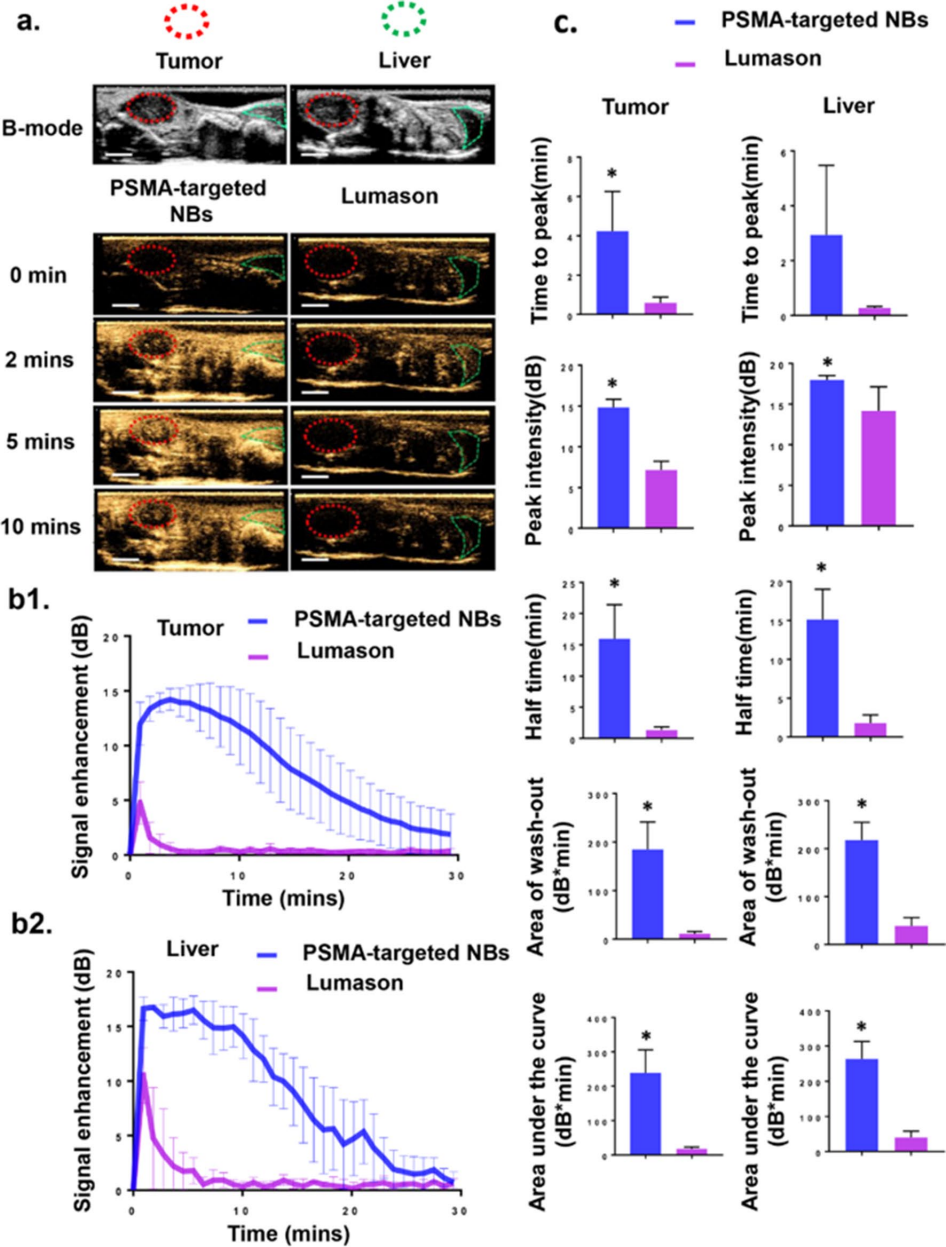
PSMA-targeted NBs provide greater tumor enhancement compared to LUMASON. (**a**) Representative ultrasonographic images of PC3pip orthotopic tumor and liver after injection of PSMA-targeted NBs and clinically available MB (LUMASON). The first and second rows showed the B-mode and CHI mode images of tumor and liver before UCAs injection. The third to the fifth rows showed the CHI images at different time points after UCAs administration. The imaging intensity in the tumor and liver from mice received PSMA-targeted NBs was apparently higher than those in animals received LUMASON at different time points. Scale bar is 0.5 cm. (**b1**) The time intensity curves (TIC) of the PC3pip orthotopic tumor after *i.v.* administration of PSMA-targeted NBs (n = 11) and LUMASON (n = 3). (**b2**) The time intensity curve (TIC) of the liver after *i.v.* administration of PSMA-targeted NBs (n = 4) and LUMASON (n = 3). (**c**) Comparison of the UCA kinetic parameters between PSMA-targeted NBs and LUMASON in the tumors or livers. Data are presented as mean ± standard deviation; *p < 0.05, PSMA-targeted NBs group *vs.* LUMASON group.

After tail vein injection of PSMA-targeted NBs (200 μL of 3.9 ± 0.282X10^11^/mL PSMA-targeted NBs) (n = 11) or LUMASON (200 μL of 1-5X10^8^/mL LUMASON MBs) (n = 3), contrast harmonic imaging (CHI) images were continuously acquired (receive frequency of 12 MHz) to determine the dynamics of the bubbles in the tumors and livers. The LUMASON dose, PSMA-targeted NB dose and imaging parameters used were optimized in our previous work^20^. It is worth noting that the nonlinear contrast imaging parameters in these studies utilized a higher frequency than typically used clinically for LUMASON (3 MHz). While these should not affect the kinetic parameters of LUMASON, they may affect the overall image quality. Under CHI mode, tumors and livers were not visible before injection of either PSMA-targeted NBs or LUMASON (Fig. 2a). A rapid enhancement started approximately 15–30 s after NB injection, and was observed first in the livers followed by tumors. The UCA kinetic parameters (Fig. 2c) were obtained from the time intensity curve (TIC) (Fig. 2b1,b2). These include time to peak, peak intensity, half time, area of wash-out and area under the curve (AUC). These parameters were compared between PSMA-targeted NBs and LUMASON both in the tumor and the liver. Although the group size for the LUMASON group was relatively small, the difference between the LUMASON group and PSMA-targeted NB group at the imaging parameters used in this study was large and a statistically significant difference was observed between the two groups. This is also consistent with previously published work^20^. The results showed that the time to peak, peak intensity, half time, area of wash-out and AUC were significantly different between PSMA-targeted NBs and LUMASON (*p* < 0.05) in the tumor, and the last four parameters were significantly different between PSMA-targeted NBs and LUMASON (*p* < 0.05) in the liver. All above indicated higher stability and longer circulation time of our PSMA-targeted NBs than for LUMASON MBs in the blood stream.

The tumor sizes in LUMASON group were between 280 and 520 mm^3^ and the tumor sizes in NB groups were from 90 to 1100 mm^3^. To make sure that the difference between LUMASON and PSMA-targeted NB was not a result of the tumor size, we split the PSMA-targeted NB groups into two groups based on tumor size: Group A (small tumor) had tumor volumes between 90 and 670 mm^3^ and Group B (big tumor) had tumor volumes between 670 and 1100 mm^3^ and compared the LUMASON group to these two groups separately. The parameters in both group A and Group B were nonetheless significantly different from those in the LUMASON group (Fig. S1). Our results confirmed that the lower peak enhancement of LUMASON was not related to the tumor size.

### Contrast agent dynamics and comparison of orthotopic prostate tumors using PSMA-targeted NBs and non-targeted NBs

**Figure 3:**
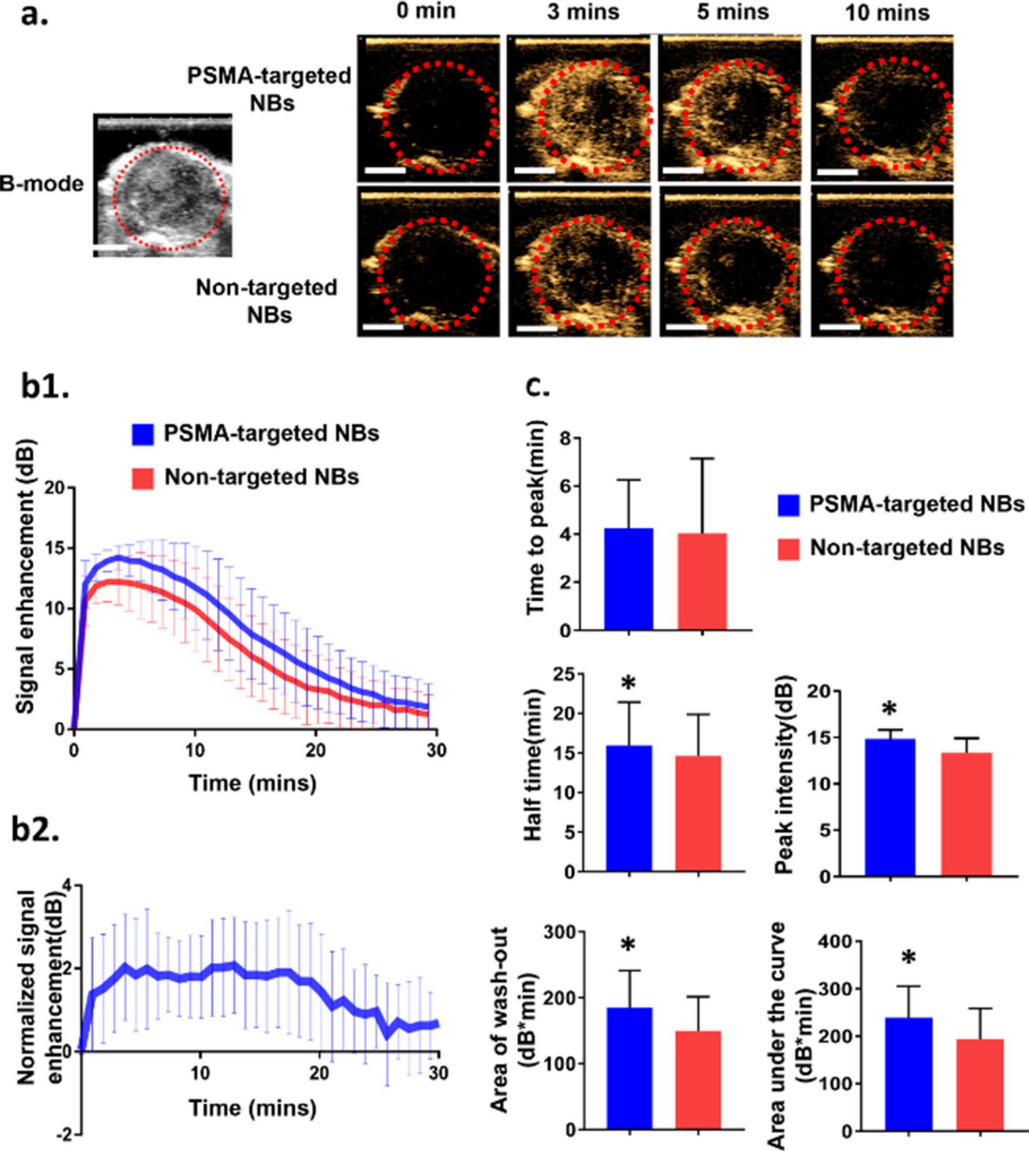
PSMA-targeted NBs provide greater tumor enhancement as compared to non-targeted NBs. (**a**) Representative ultrasonographic images of PC3pip orthotopic tumor after injection of PSMA-targeted NBs and non-targeted NBs (n = 11). The first and second columns showed the B-mode and CHI mode images of tumor before UCAs injection, respectively. The third to the fifth columns showed the CHI images at different time points after UCAs administration. Scale bar is 0.5 cm. (**b1**) The time intensity curves (TIC) of the PC3pip orthotopic tumor after *i.v.* administration of PSMA-targeted NBs and non-targeted NBs. (**b2**) US signal obtained from non-targeted NBs measurements were used to normalize the signal from PSMA-targeted NBs. The normalized signal enhancement means (Intensity_PSMA-targeted NBs_ – Intensity_non-targeted NBs_) (**c**) Comparison of the UCA kinetic parameters between PSMA-targeted NBs and non-targeted NBs in tumor. Data are presented as mean ± standard deviation(n = 11) ; *p < 0.05 targeted NB *vs.* non-targeted NBs.

To evaluate the selective imaging ability of PSMA-targeted NBs toward prostate tumor, non-targeted NBs were used as a comparison. US scans with both bubble formulations were performed under identical conditions, and the average results of 11 nude mice bearing PC3pip orthotopic tumors were reported. First, the PC3pip tumors were localized in B-mode, and then we switched to contrast mode. Tumors were not visible in the contrast mode before bubble injection (Fig. 3a). Continuous contrast mode US was performed to monitor the bubble dynamic in the tumors after *i.v.* injection of PSMA-targeted NBs or non-targeted NBs. The bubble kinetics obtained from the time intensity curve (TIC) (Fig. 3b1), which includes time to peak, peak intensity, half time, area of wash-out and area under the curve (AUC), were compared among PSMA-targeted NBs and non-targeted NBs in the PSMA ( +) PC3pip orthotopic tumors (Fig. 3c). Significant differences in peak intensity (*p* = 0.0001), half time (*p* = 0.0056), area of wash-out (*p* = 0.0092) and area under the curve (*p* < 0.0001) were measured between PSMA-targeted NBs and non-targeted NBs. US signal obtained from non-targeted NBs measurements was used to normalize the signal from PSMA-targeted NBs. The normalized TIC showed that the average intensity from PSMA-targeted NBs was always higher than non-targeted NBs at different time points (Fig. 3b2). Since the tumor sizes used in this study varied from 90 to 1100 mm^3^, we also divided the animals into two cohorts: Group A (small tumor, 90–670 mm^3^, n = 7) and Group B (big tumor, 670–1100 mm^3^, n = 4) and compared the parameters of PSMA-targeted and non-targeted NBs (Fig. S2). In Group A with small tumors, significant differences in peak intensity and area under the curve were observed. In Group B with big tumors, significant differences in peak intensity, area under the curve and half time were seen. The TIC of individual mice also showed differences between PSMA-targeted NBs and non-targeted NB in 10 out of 11 mice (Fig. S3). Although inter-animal viability was observed, the overall results between the two groups were similar. Altogether, our data indicated higher stability and longer circulation time of our PSMA-targeted NBs than that of non-targeted NBs in the blood stream.

### Contrast agent dynamics and comparison based on different tumor sizes

**Figure 4:**
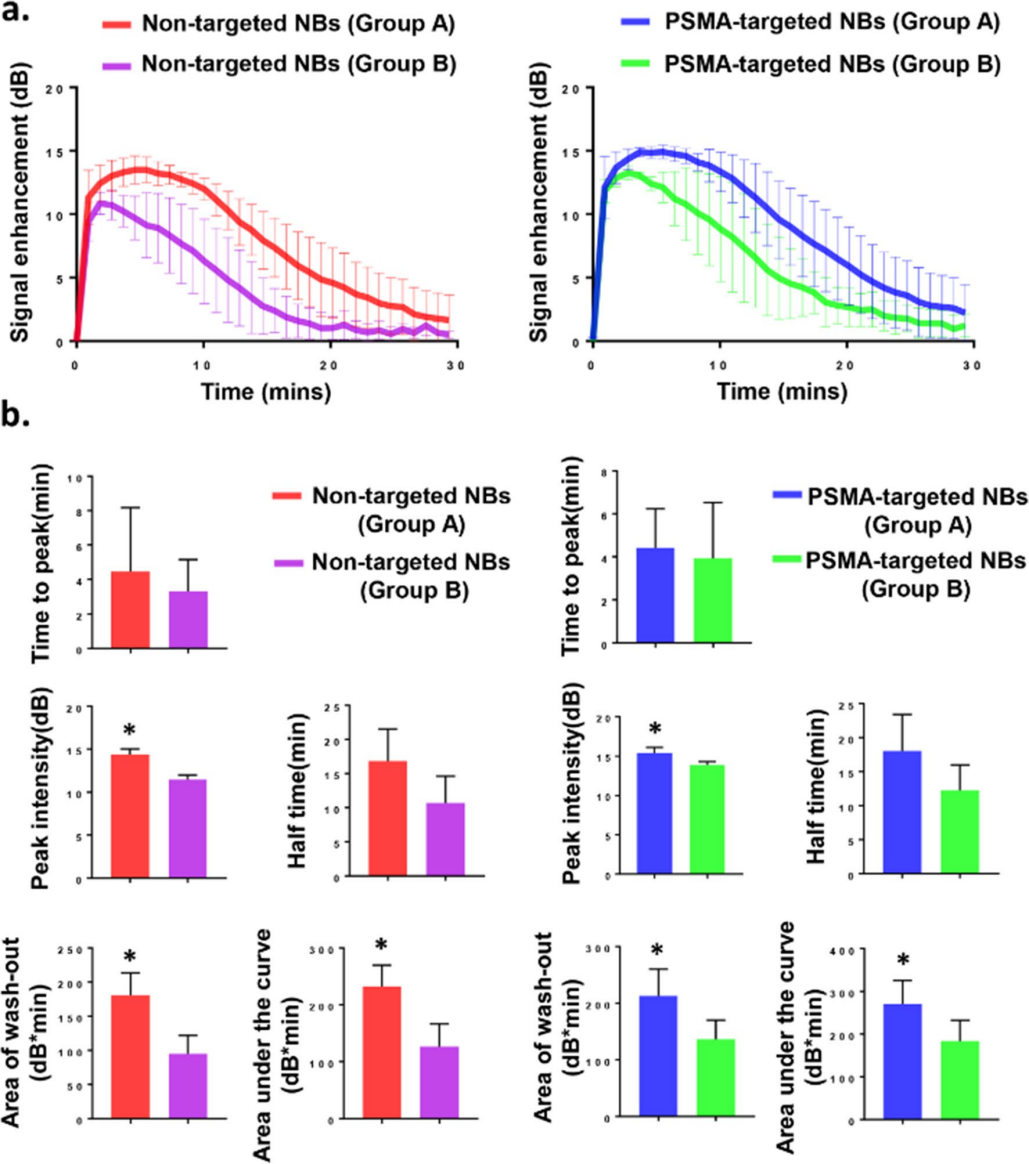
PSMA-targeted NBs and non-targeted NBs provide more tumor enhancement in small tumors (Group A) as compared to that in large tumors (Group B). (**a**) The time intensity curves (TIC) of tumor after *i.v.* administration of PSMA-targeted NBs and non-targeted NBs in Group A (n = 7) and Group B (n = 4). (**b**) Comparison of the UCA kinetic parameters between Group A (n = 7) and Group B (n = 4). Data are presented as mean ± standard deviation; *p < 0.05, group A *vs*. group B.

Due to apparent variability in the dynamics of PSMA-targeted NBs and non-targeted NBs depending on the size of the tumor, tumors were separated into two groups: Group A had tumor volumes between 90 and 670 mm^3^ (n = 7), and Group B had tumor volumes between 670 and 1100 mm^3^ (n = 4). The UCA kinetic parameters (Fig. 4b) were obtained from the time intensity curve (TIC) (Fig. 4a). As tumor sizes increased, the peak intensity, area of wash-out, and total area under the curve were significantly different between Group A and Group B (Fig. 4b). As shown in Fig. 3a, some part of the tumor didn’t fill well after bubble injection in Group B, and the signal was heterogeneous in B-mode; while in Group A, the signal was relatively homogeneous both in B-mode and contrast mode (Fig. 2a).

### PSMA-targeted NBs are retained in the orthotopic prostate tumor after bubble clearance from circulation

**Figure 5:**
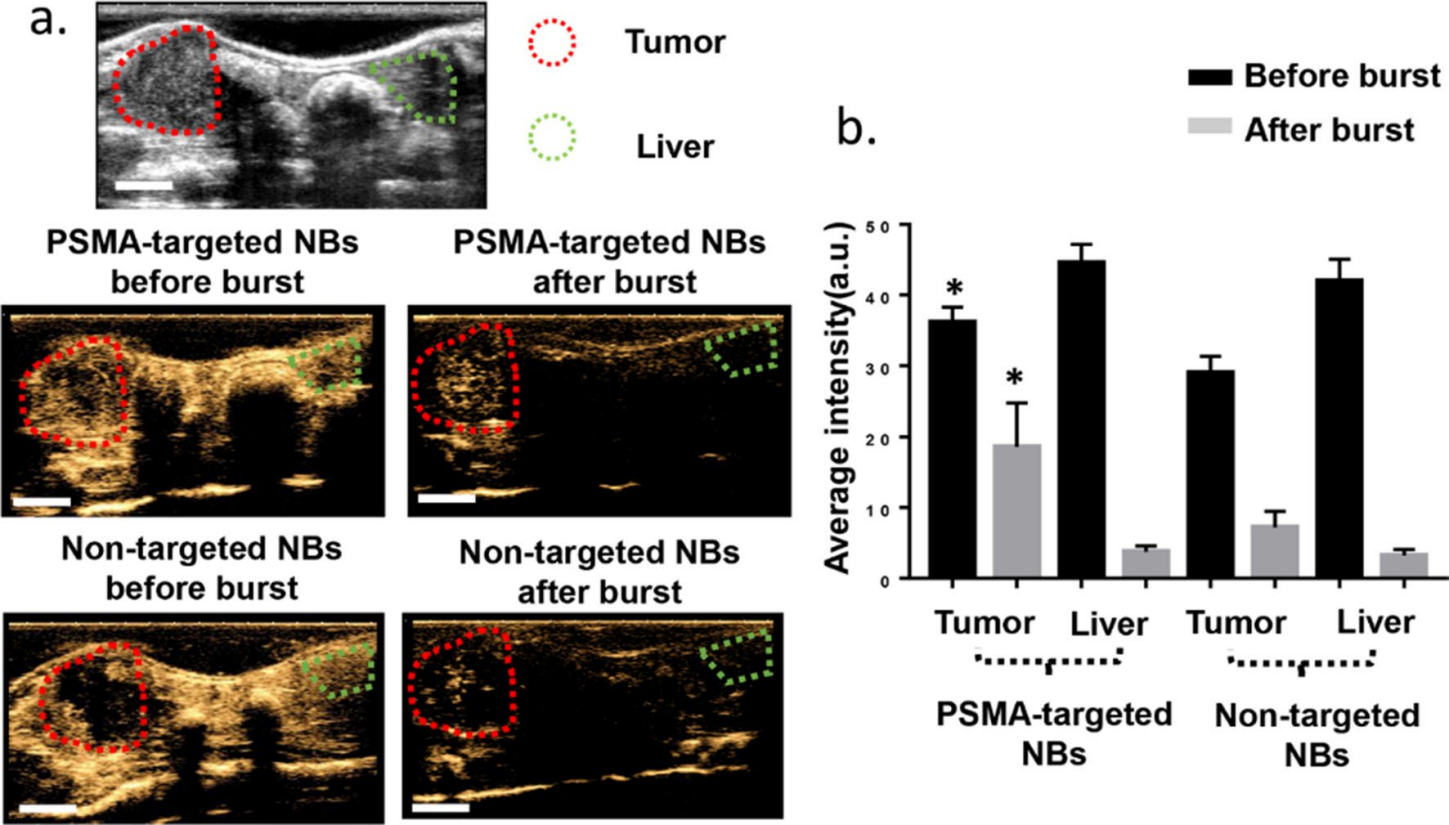
PSMA-targeted NBs enable prolonged imaging and greater US signal in PSMA-positive PC3pip tumors after removing nanobubbles from the circulation. (**a**) The first row showed the B-mode image of the tumor and liver before injection. The second row showed the CHI of the tumor and liver before bubble burst. The third row showed the CHI of the tumor and liver after bubble burst. Scale bar is 0.5 cm. (**b**) The average signal intensities of bubbles in the tumor and liver before and after burst. Data are presented as mean ± standard deviation, *p < 0.05, targeted group *vs.* non-targeted group, n = 4.

The bubble burst studies were performed in 4 additional mice bearing orthotopic PC3pip tumor at the size of 300–800 mm^3^ and the average results were reported in Fig. 5. The series of images in Fig. 5a showed the CHI images before and after bursting the bubbles in circulation via repeated high intensity pulses applied to the liver. Quantitative analysis of the enhancement (Fig. 5b) showed a 47.9 ± 18.6% reduction in signal after clearance in the PC3pip tumors with targeted NBs, compared to 74.8 ± 8.9% with non-targeted NBs in tumor and 92.2 ± 2.4% in the liver. These data showed significantly higher peak enhancement in the tumors enhanced using PSMA-targeted NBs compared to non-targeted NBs. Most importantly, following the clearance of circulating NBs via repeated high-intensity pulses, the signal intensity in tumors enhanced using PSMA-targeted NBs remained significantly higher compared to non-targeted NBs. This suggests significant NB retention in PSMA-expressing PCa cells. In contrast to the tumors, the signal intensity in the liver was similar with both PSMA-targeted NBs and non-targeted NBs before the burst and was nearly completely eliminated after clearance for both agents. It is important to note that the tumor region in these studies was not exposed to constant insonation (in contrast with the tumors used to generate the TIC) which preserved bubble echogenicity for a longer time. This is likely to have magnified the differences between targeted and untargeted NBs seen in this experiment. This data shows, for the first time, significant extravascular retention of PSMA-targeted NBs in PSMA-positive PC3pip tumor parenchyma (likely within the tumor cells) after clearance of NBs from circulation in live mice.

### Immunohistochemical analysis

**Figure 6:**
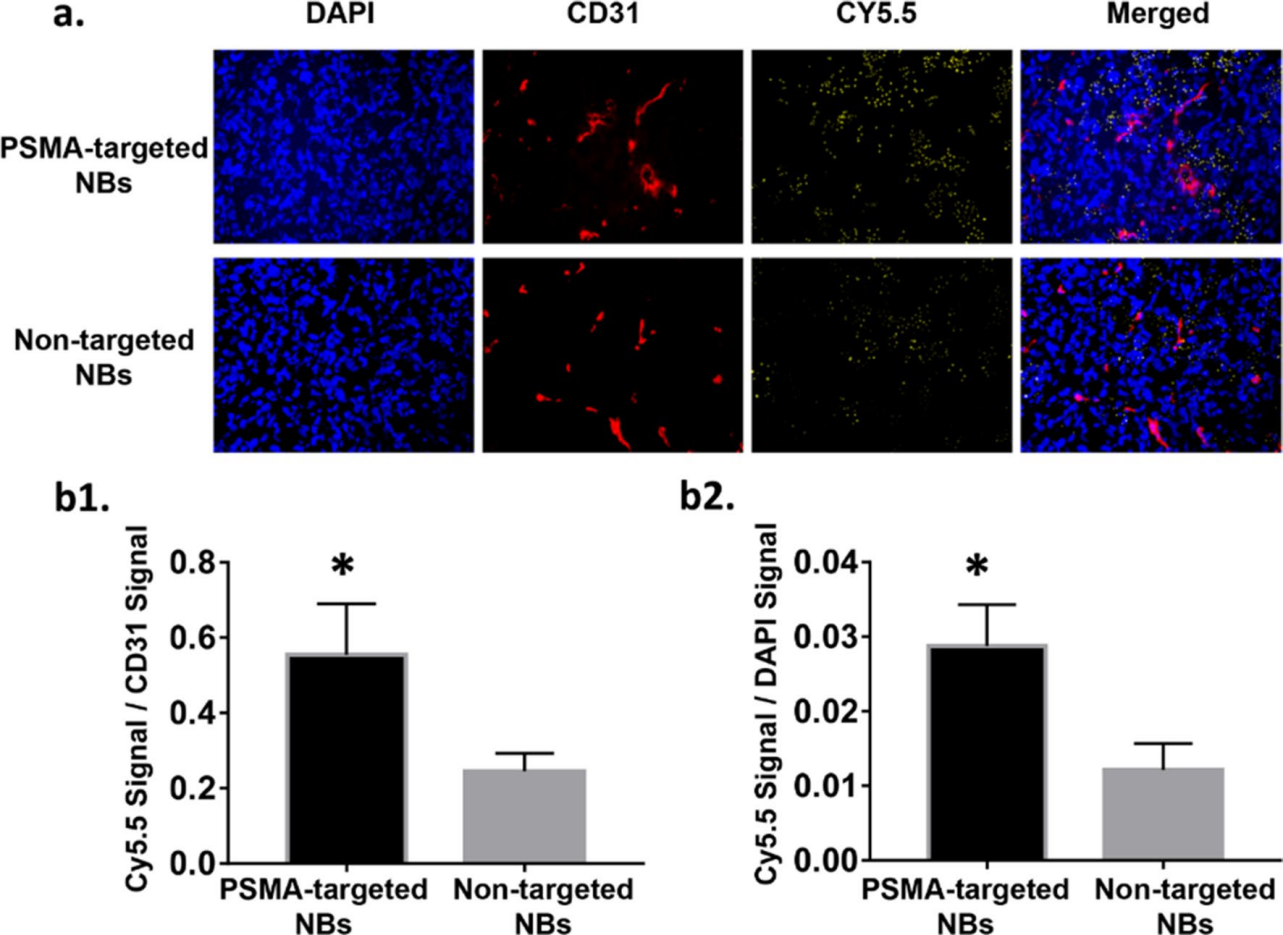
Histological images of Cy5.5 and CD31 signal in tumors treated with PSMA-targeted NBs or non-targeted NBs after perfusion. (magnification:20x) (**a**) Cy5.5 and CD31 signals in the tumor after perfusion. N = 3 for both PSMA-1-targeted group and non-targeted group. (**b1**) Quantification of fluorescence ratio (total bubbles fluorescence/vessels fluorescence per field). Data are presented as mean ± standard deviation; *p < 0.05, targeted group *vs.* non-targeted group, n = 3. (**b2**) Quantification of fluorescence ratio (total bubbles fluorescence/cells fluorescence per field). Data are presented as mean ± standard deviation; *p < 0.05, targeted group *vs.* non-targeted group, n = 3.

To further validate that PSMA-targeted NBs could extravasate into the tumor matrix, the bubbles were labeled with a fluorescent dye Cy5.5 and injected to a new set of animals bearing orthotopic PC3pip tumor. Ten minutes after injection, mice underwent a cardiac flush perfusion procedure with cold PBS to remove circulating bubbles and tumors were harvested for histological analysis. CD31 staining was used to visualize the tumor vessels. The fluorescence in the vessels and cells was used to normalize the bubbles signal per field. Histological images showed that Cy5.5 signal of PSMA-targeted NBs group was found outside of tumor capillaries and deep in the parenchyma (Fig. 6a), which provided strong evidence of bubble extravasation and subsequent interstitial penetration. The NB fluorescence ratio (quantification of fluorescence ratio from total bubbles fluorescence/vessels fluorescence and total bubbles fluorescence/cells fluorescence per field) in PSMA-targeted NBs group was significantly higher than that in non-targeted NBs group (Fig. 6b1,b2), which confirmed that PSMA-targeted NBs not only can extravasate into the tumor but also can be trapped within the tumor.

## Discussion

It is estimated that there will be around 500,000 annual deaths due to PCa by 2030^26^. The earlier PCa is detected, the more likely it can be adequately treated, thus decreasing the mortality rate^27–29^. There is an urgent need for an effective method to diagnose the disease early.

Contrast enhanced-US has been investigated in several studies for diagnosis of PCa. Frausher et al*.* showed that the use of a microbubble ultrasound contrast agent for transrectal color Doppler targeted biopsy significantly improved the detection of PCa compared with systematic biopsy following conventional gray-scale ultrasonography (*p* < 0.001)^30^. Halpern et al*.* showed that future applications of MB agents could likely expand to include the staging of PCa and the monitoring of response to therapy^31^. Sanna et al*.* reported the first model of polymeric MBs targeted to PSMA in 2011^32^. Experiments were performed in vitro because MBs only circulate in the bloodstream, which is their main limitation in tumor-targeted imaging^33^. In recent years, NBs have been applied in extravascular ultrasonic imaging^33^. Because of their small size, NBs do not get trapped in the blood pool after intravenous injection. Cai et al*.* and Wu et al*.* among others have shown that NBs with phospholipid-shell and gas-core had longer tumor enhancement time compared to MB such as SONOVUE or DEFINITY in vivo due to enhanced permeation and retention (EPR) effect^18,34^. They also found that the addition of DSPE-PEG2000 lipids could prevent NBs from being cleared by the reticuloendothelial system, thus increasing their retention. Similar to MBs, another advantage of NBs is that they can undergo surface modification to increase tumor selectivity and enhance tumor theragnostic. Ligands and receptors can be incorporated to the surface of NBs for specific delivery of therapeutic agents^35^. The goal of this study was to formulate a novel targeted, nanoscale ultrasound contrast agent to detect PSMA ( +) PCa in a clinically relevant orthotopic model. Our previous study in a flank tumor model has already examined the kinetics of PSMA-targeted NBs and non-targeted NBs, and histological findings confirmed that PSMA-targeted NBs can specifically recognize the tumors with PSMA expression^20^. In this study, significant differences were observed in peak intensity, half time, area of wash-out and area under the curve between PSMA-targeted NBs and non-targeted NBs for orthotopic tumors (Fig. 3). Comparing the results obtained from orthotopic the tumor model to previous work in the flank tumor model^20^, the signal difference between PSMA-targeted NBs and non-targeted NBs in orthotopic PC3pip tumor was less obvious than that in flank PC3pip tumor. More specifically, while the total AUC was comparable for PSMA-NBs between the two models, the untargeted NB AUC and especially the washout AUC were increased in the orthotopic model. Western blot studies showed that flank PC3pip tumor and orthotopic PC3pip had similar level of PSMA expression (Fig. S4), therefore, the difference was not due to different levels of PSMA expression. We hypothesize that this difference may be a consequence of three factors: 1) variability in vascular density and vascular permeability in the two tumor models, as well as 2) the overall tumor burden and 3) differences in cellular density and central necrosis. Differences between tumor microenvironments between flank and orthotopic tumors are known to affect these factors. Specifically, it has been reported that orthotopic PC3 tumors have higher vascular volume and permeability than flank PC3 tumors^36^. The higher vascular permeability of the orthotopic tumor may enable greater extravasation of all nanobubbles by the enhanced permeability and retention (EPR) effect. It is thus possible that the enhanced permeability will lead to increased uptake of both PSMA-targeted NBs and non-targeted NB; therefore, the difference of NB accumulation in orthotopic tumors between PSMA-targeted NBs and non-targeted NBs accumulation is smaller than that in flank tumor models. This is also reflected by the higher overall area under the time intensity curve (193.7 ± 64.38 dB*min in orthotopic model *vs* 130.4 ± 50.11 dB*min in flank model) and the washout AUC for the non-targeted NBs (149.5 ± 52.19 dB*min in orthotopic model *vs* 116.51 ± 25.61bB*min in flank model), which is seen in this model versus the flank tumors^20^. If the necrosis and cellular density are higher, this would also result in greater retention of all bubbles, thus reducing the washout of untargeted ones. In general, there are many potential differences between these models, that can result in the specific changes in the TIC. This is partially why using NB contrast enhanced ultrasound may provide some insight into nanoparticle transport in tumors. The average tumor size in the flank tumor was around 125 mm^3^, while the average tumor size in the orthotopic tumor was around 500 mm^3^. Stratifying tumors into large and small cohorts illustrated significant difference in peak intensity, area of wash out and area under the curve (Fig. 4), indicating that tumor burden is also a factor that affects the kinetics. Inter-animal variability was also observed in animals (Fig. S3). When normalized to each individual animal (as shown in Fig. 3B2) the difference in enhancement is higher. It is likely that orthotopic tumors are more heterogeneous than flank tumors, thus leading to reduced differences on average.

In this study, a bubble burst study was used to detect the signal in tumor after bursting the circulating bubbles, which indicated that PSMA-targeted NBs were retained in the tumor to a greater extent than non-targeted NBs (Fig. 5). In addition, we also found that kinetics and tumor distribution of our NBs varied depending on tumor size/stage. Histology studies of the small tumors and big tumors found that the center of the big tumors was more necrotic than that of the small tumor (Fig. S5). This was also consistent with a previous literature report about the heterogeneity of the EPR effect^37^ and prior studies showing that larger murine tumors (e.g., 1–2 cm in diameter) tend to contain more necrotic tissues or highly hypovascular areas^38^. In general, tumor vessels are immature, irregular shaped and lack a normal hierarchy of vessels types^39^. Gee et al*.* indicated that most vessels in transplanted mouse tumors were newly formed because a relatively low fraction of pericyte-covered vessels appeared^40^. Russo et al*.* showed that most tumors developed starting from an avascular phase followed by sprouting and tube formation, and tumor vascular organization may be completely different depending on its location and whether it was growing, repressing, or relapsing^13^. These also explained the possible reason that the differences between the smaller and larger tumors in our study.

Previously Wang et al*.* developed a PSMA monoclonal antibody-modified nanoscale UCAs and showed they could specifically bind to PCa cells in vitro and could significantly increase peak intensity and duration of contrast enhancement than blank NBs in transplanted prostate tumors^41^. However, the biotin-avidin method may not be clinically relevant^42^. In contrast, the targeting ligand, PSMA-1, in our study is a peptide-based highly negatively charged PSMA ligand, which can be used in clinical research and also can be easily synthesized^43^. Fan et al*.* conjugated their nanobubbles with anti-PSMA nanobody and showed that nanobody-coated nanobubbles could enhance the diagnostic value of ultrasound in PCa^44^. Their average particle diameter was 487.60 ± 33.55 nm, which was significantly smaller (*p* = 0.003) than their previously produced nanobubbles that carried PSMA monoclonal antibodies (644.30 ± 55.85 nm)^44^. In contrast, the average diameter of our PSMA-targeted NBs was 277 ± 11 nm. The smaller size of our NBs should achieve better tumor penetration than bigger size bubbles. Smaller size of particles has been shown to improve the biodistribution and the enhanced permeability and retention effect of nanoparticles in a murine xenograft tumor model^45^. Overall, the current data suggest that: 1) echogenic nanobubbles labeled with a high affinity ligand to PSMA are considerably more stable in vivo and show greater differences in kinetics between clinical MBs and non-targeted NBs; 2) the NBs appear to have distinct kinetics and retention in tumors of different sizes. This could be a promising area of future investigation, as a means of staging and potentially grading tumors using the same agents.

BR55 are promising microbubbles targeting VEGFR2, which are currently undergoing clinical trials to determine specificity and sensitivity of detection of prostate, breast and ovarian tumors angiogenesis for enhancement of contrast^46^. BR55 is confined to blood stream, and cannot penetrate the tumor parenchyma. This limits the overall utility of BR55 to vascular targets and makes most cellular and tumor microenvironment biomarkers for PCa and other cancer inaccessible. Compared to BR55, PSMA-targeted NBs have the potential to be more specific as they have been shown to extravasate and be taken up into PCa cells via receptor-mediated endocytosis^47^. This can potentially increase specificity and sensitivity of detection for an extended time. In addition, our data have shown that our PSMA-targeted NBs have higher stability and longer circulation time in the blood stream in mice than clinically used LUMASON MBs, providing longer imaging time. This is, of course, dependent on the imaging parameters. For example, in this and other NB-studies^20^, we have utilized frequencies higher than the typical 3 MHz used in clinical scans, which may limit sensitivity of detection of microbubbles such as LUMASON.

## Conclusion

This work demonstrates expanded capabilities of ultrasound molecular imaging which can be used to examine biomarker expression on cancer cells in the tumor. Retention in tumors was enhanced by a targeting ligand, and the process was detected with US. Because of the prolonged retention compared to non-targeted NBs, PSMA-targeted NBs have a better value in the diagnosis of orthotopic PSMA ( +) prostate tumors in mice. This may provide methods for relevant studies on targeted ultrasound NBs and prove that targeted NBs have the potential to become a more sensitive detection tool in the diagnosis of PSMA ( +) PCa cancer. Finally, targeted NBs have the potential to be used as contrast agents to inform PCa biopsies and potentially can be developed for image guided PCa biopsy.

## Materials and methods

### Preparation of PSMA-targeted and non-targeted NB

PSMA-targeted NB (10 mg/mL) was prepared as previously reported^19,20^ by first dissolving a mixture of lipids comprising of 1,2-dibehenoyl-sn-glycero-3-phosphocholine (C22, Avanti Polar Lipids Inc., Pelham, AL), 1,2 Dipalmitoyl-sn-Glycero-3-Phosphate (DPPA, Corden Pharma, Switzerland), 1,2-dipalmitoyl-sn-glycero-3-phosphoethanolamine (DPPE, Corden Pharma, Switzerland), and 1,2-distearoyl-snglycero-3-phosphoethanolamine-N-[methoxy(polyethylene glycol)-2000] (ammonium salt) (DSPE-mPEG 2000, Laysan Lipids, Arab, AL) into propylene glycol (0.1 mL, Sigma Aldrich, Milwaukee, WI) by heating and sonicating at 80 °C until all the lipids were dissolved. Mixture of glycerol (0.1 mL, Acros Organics) and phosphate buffered saline (0.8 mL, Gibco, pH 7.4) preheated to 80 °C was added to the lipid solution. The resulting solution was sonicated for 10 min at room temperature. DSPE-mPEG-PSMA (25 µL in 1 mg/mL PBS) was added. The solution was transferred to a 3 mL-headspace vial, capped with a rubber septum and aluminum seal, and sealed with a vial crimper. Air was manually removed with a 30 mL-syringe and was replaced by injecting octafluoropropane (C_3_F_8_, Electronic Fluorocarbons, LLC, PA) gas. The phospholipid solution was then activated by mechanical shaking with a VialMix shaker (Bristol-Myers Squibb Medical Imaging Inc., N. Billerica, MA) for 45 s. PSMA-targeted NBs were isolated from the mixture of foam and microbubbles by centrifugation at 50 rcf for 5 min with the headspace vial inverted, then 200 µL PSMA-targeted NB solution was withdrawn from a fixed distance of 5 mm from the bottom with a 21G needle. Similar preparation was carried out for non-targeted NB but without the addition of DSPE-mPEG-PSMA^20^.

### Size, concentration, and surface charge of NBs

The size distribution and concentration of PSMA-targeted NBs and non-targeted NBs were characterized with a Resonant Mass Measurement (ARCHIMEDES, Malvern Panalytical) equipped with a nanosensor capable of measuring particle size between 50 and 2000 nm^19,20^. The NB solution was diluted with PBS (500x) to obtain an acceptable limit of detection (< 0.01 Hz) and coincidence (< 5%). During the sample measurement, NB solution was loaded at 2 psi for 120 s and analyzed at 5 psi. Surface charge of the diluted NB solution (500X) was measured with an Anton Paar Litesizer 500.

### Animal models

Animals were handled according to a protocol approved by the Institutional Animal Care and Use Committee (IACUC) at Case Western Reserve University and were in accordance with all applicable protocols and guidelines in regard to animal use. Four to six-week old male athymic Balb/c nude mice were purchased from Case Western Reserve University animal research center and housed in the small animal imaging center, an approved Animal Resource Center. All animals received standard care: Ad libitum access to food and water; 12/12 light/dark cycle; Species appropriate temperature and humidity; Environmental enrichment and group housing whenever possible; Standard cage sanitization; and solid bottom caging. Mice were anesthetized with inhalation of 1–2% isoflurane with 0.5–1 L/min oxygen. A 28 1/2-gauge insulin needle was inserted into ventral prostate gland to deliver 10 µL PSMA ( +) PC3pip cells suspended in PBS (phosphate buffered saline). A well-localized bleb within the injected prostate lobe is a sign of a technically satisfactory injection. Animals were observed every other day until tumors reached at about 3-5 mm in diameter, and then used for imaging studies.

### Pharmacokinetic study

Animals were used in the study 10 days after inoculation when the tumor diameter reached 3–5 mm. The pharmacokinetics of the NBs were monitored by APLIXG SSA-790A Toshiba Medical Imaging Systems (Otawara-Shi, Japan) using the ultrasound probe PLT-1204BT. After mice were anesthetized with 1–2% isoflurane with 0.5–1 L/min oxygen, each mouse was placed in the face-up position, and the ultrasound probe (PLT-1204BT) was placed longitudinally to the axis of the animal body to visualize the ultrasound images of the PC3pip orthotopic tumors. To compare contrast enhanced ultrasound images with the same tumor in the same mouse (n = 11), 200 μL of either PSMA-targeted NBs (3.9 ± 0.282X10^11^/mL) or non-targeted NBs (4.0 ± 0.245X10^11^/mL) were administrated via tail vein. Before NB injections, the images were acquired in raw data format for 5 s. After injection of NBs, contrast harmonic imaging (CHI) was used to image the change of tissue contrast density (CHI, frequency 12.0 MHz; MI, 0.1; dynamic range, 65 dB; gain, 70 dB; imaging frame rate, 0.2 frames/s). Mice were imaged continuously for 30 min. The remaining NBs were burst by repeated flash replenish and then the same mouse received non-targeted NBs or PSMA-targeted NBs 30 min later^20^. LUMASON (200 μL, 1-5X10^8^/mL, sulfur hexafluoride lipid-type A microspheres, Bracco Diagnostics Inc.) was tested in the other 3 mice. LUMASON was prepared according to the protocol provided by the manufacturer. The raw data were processed with software provided by the scanner manufacturer. The acquired linear raw data images were processed with CHI-Q quantification software (Toshiba Medical Imaging Systems, Otawara-Shi, Japan). Regions of interest (ROIs) were drawn outlining the areas of the tumor and the liver. The signal intensity in each ROI as a function of time (time-intensity curve—TIC) was calculated and exported to Excel. To analyze the decay of ultrasound contrast, the baseline was subtracted from TIC^20^.

### Bubble burst study

Mice received 200 μL of NBs (3.9 ± 0.282X10^11^/mL) via tail vein injection. Five minutes after contrast agent injection, images were taken in 4 different planes including tumor and liver in the same field of view, and then 25-times flashing were used in different positions from the liver plane to the heart plane in order to burst all the NBs left in the circulation. After that, images were taken again in 4 different planes including the tumor and the liver in the same field of view using contrast-mode imaging. The average intensity was analyzed by Image J. The experiment was repeated in 4 nude mice bearing PC3pip orthotopic tumors.

### Histological analysis

Animals were divided into 3 groups: PSMA-NB (n = 3), plain-NB (n = 3), and no contrast control (n = 3). The method was the same as our previous study^20^. Mice received either 200 μL of contrast material or PBS alone via tail vein. Ten minutes after contrast agent injection, PBS perfusion was performed with 50 mL PBS though left ventricle. After perfusion tumors and livers were harvested and embedded in optimal cutting temperature compound (OCT Sakura Finetek USA Inc., Torrance, CA). The tissues were cut into 9 µm slices, and then CD31 staining was performed to visualize the tumor vessels. Briefly, tissues were washed 3 times with PBS and incubated with protein blocking solution that contain 0.5% Triton X-100 (Fisher Scientific, Hampton, NH). Then tissues were incubated in 1:250 diluted CD31 primary antibody (Fisher Scientific, Hampton, NH) for 24 h at 4℃. After washed with PBS, tissues incubated with Alexa 568 tagged secondary antibody (Fisher Scientific, Hampton, NH) for one hour and stained with DAPI (Vecor Laboratories, Burlingame, CA) using standard techniques. Then fluorescence images were observed under Leica DM4000B fluorescence microscopy (Leica Microsystem Inc, Buffalo Grove, IL) and then analyzed by Image J.

### Statistical analysis

Origin 2018 (Origin Lab Corporation, Northampton, MA, USA) and Excel Software (Microsoft Corporation, Henderson, NV, USA) were used to calculate the ultrasound parameters. GRAPH PAD PRISM 7 (GraphPad Software, La Jolla, CA, USA) was used to plot histograms and the curve with non-linear regression. All statistical analyses were performed by Statistical Package for Social Science (SPSS) 22.0 software (IBM Corporation, Armonk, NY, USA). All ultrasound parameters between PSMA-targeted NBs and non-targeted NBs were compared and analyzed using the paired-sample Student’s *t*-test, and other comparison in this paper used unpaired Student’s *t*-test. A *p* value < 0.05 was considered statistically significant. All data are presented as mean ± SD (standard deviation).

### Ethical approval

Animals were handled in compliance with the ARRIVE guidelines and in according to a protocol approved by the Institutional Animal Care and Use Committee (IACUC#150,033, approved on March 15, 2015) at Case Western Reserve University. The experiments were carried out in accordance with all applicable protocols and guidelines in regard to animal use.

## Supplementary Information

Supplementary Figures..

## Data Availability

The authors declare that all available data are present in the manuscript.
