# Supplementary material for: Molecular imaging of orthotopic prostate cancer with nanobubble ultrasound contrast agents targeted to PSMA
Source: Sci Rep. 2021 Feb 25;11:4726. doi: 10.1038/s41598-021-84072-5 (PMC7907080; doi:10.1038/s41598-021-84072-5)
Supplement: Supplementary file 1 — Supplementary Figures.. [file 41598_2021_84072_MOESM1_ESM.pptx]

## Slide 1
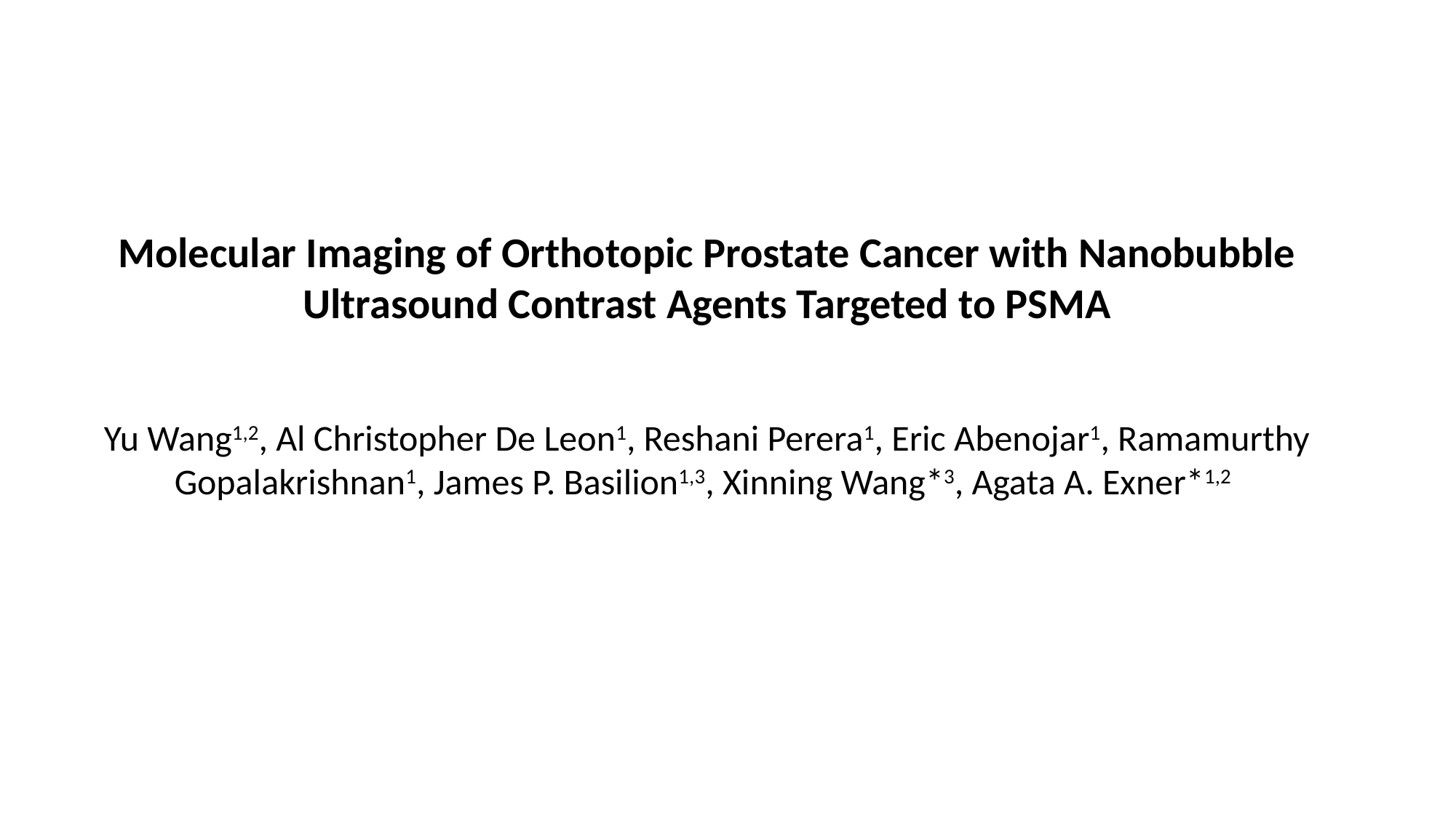

Molecular Imaging of Orthotopic Prostate Cancer with Nanobubble Ultrasound Contrast Agents Targeted to PSMA
Yu Wang1,2, Al Christopher De Leon1, Reshani Perera1, Eric Abenojar1, Ramamurthy Gopalakrishnan1, James P. Basilion1,3, Xinning Wang*3, Agata A. Exner*1,2

## Slide 2
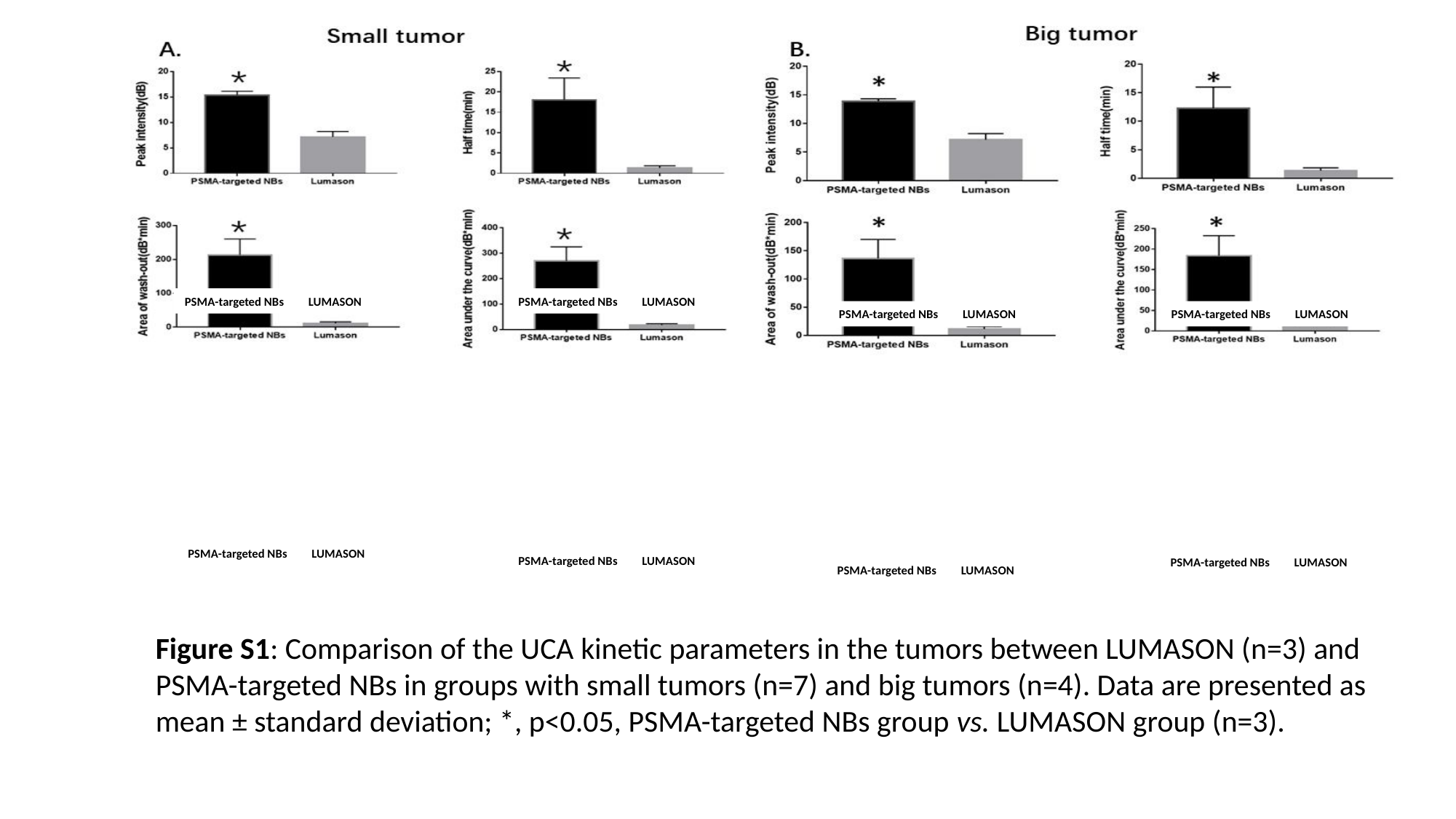

PSMA-targeted NBs LUMASON
PSMA-targeted NBs LUMASON
PSMA-targeted NBs LUMASON
PSMA-targeted NBs LUMASON
PSMA-targeted NBs LUMASON
PSMA-targeted NBs LUMASON
PSMA-targeted NBs LUMASON
PSMA-targeted NBs LUMASON
Figure S1: Comparison of the UCA kinetic parameters in the tumors between LUMASON (n=3) and PSMA-targeted NBs in groups with small tumors (n=7) and big tumors (n=4). Data are presented as mean ± standard deviation; *, p<0.05, PSMA-targeted NBs group vs. LUMASON group (n=3).

## Slide 3
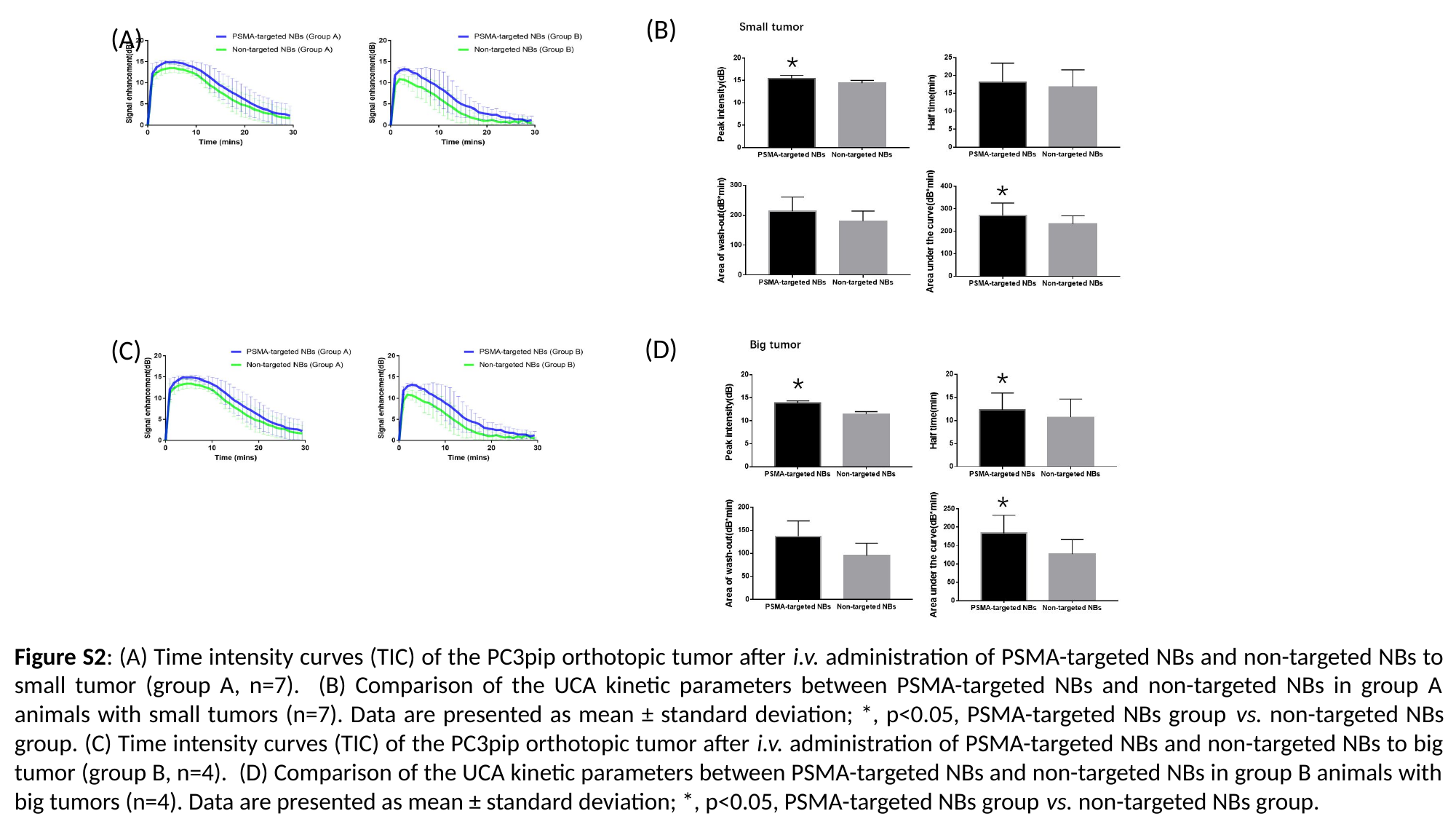

(B)
(A)
(D)
(C)
Figure S2: (A) Time intensity curves (TIC) of the PC3pip orthotopic tumor after i.v. administration of PSMA-targeted NBs and non-targeted NBs to small tumor (group A, n=7). (B) Comparison of the UCA kinetic parameters between PSMA-targeted NBs and non-targeted NBs in group A animals with small tumors (n=7). Data are presented as mean ± standard deviation; *, p<0.05, PSMA-targeted NBs group vs. non-targeted NBs group. (C) Time intensity curves (TIC) of the PC3pip orthotopic tumor after i.v. administration of PSMA-targeted NBs and non-targeted NBs to big tumor (group B, n=4). (D) Comparison of the UCA kinetic parameters between PSMA-targeted NBs and non-targeted NBs in group B animals with big tumors (n=4). Data are presented as mean ± standard deviation; *, p<0.05, PSMA-targeted NBs group vs. non-targeted NBs group.

## Slide 4
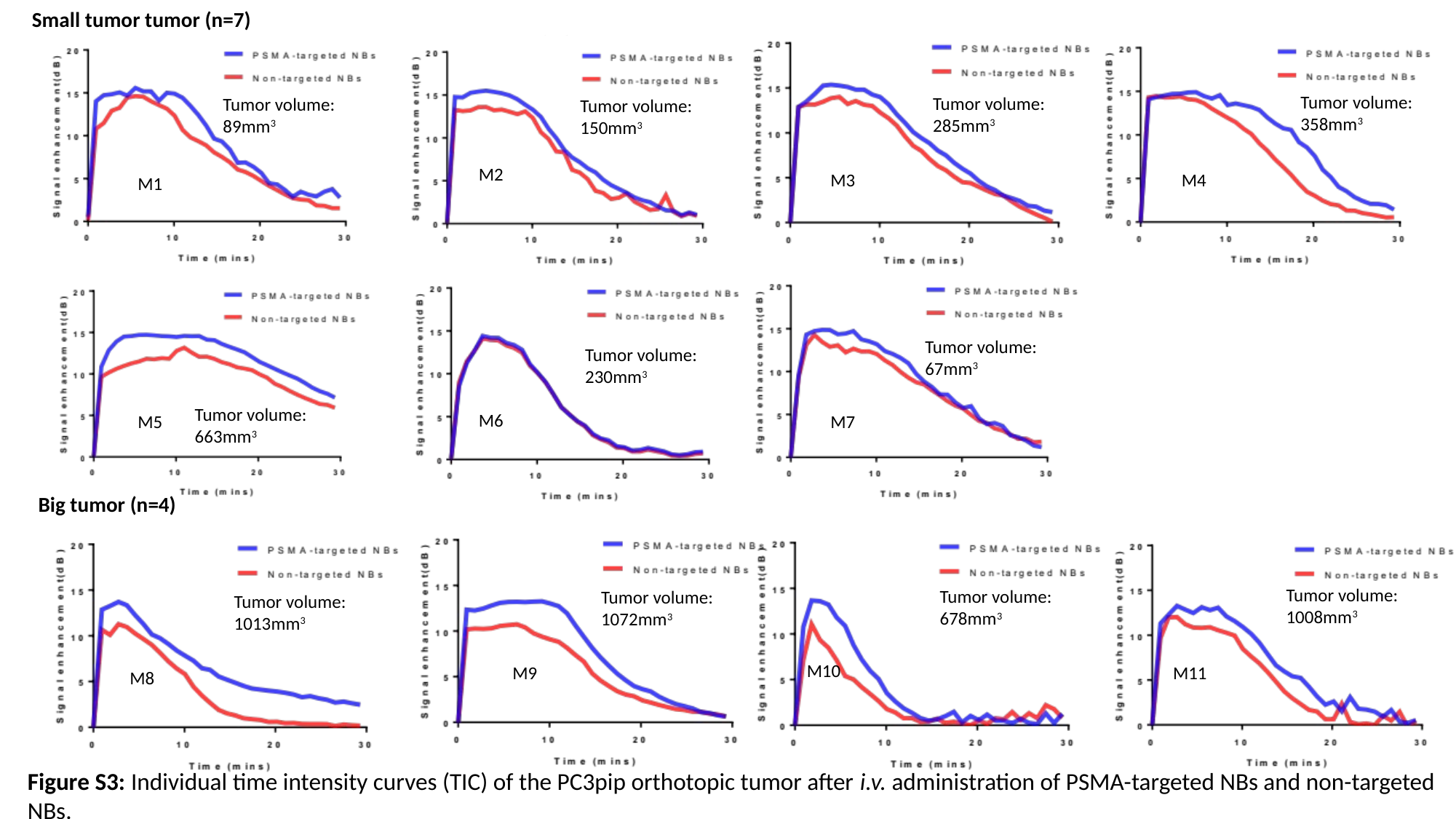

Small tumor tumor (n=7)
Tumor volume: 358mm3
Tumor volume: 285mm3
Tumor volume: 89mm3
Tumor volume: 150mm3
M2
M3
M4
M1
Tumor volume: 67mm3
Tumor volume: 230mm3
Tumor volume: 663mm3
M6
M5
M7
Big tumor (n=4)
Tumor volume: 1008mm3
Tumor volume: 678mm3
Tumor volume: 1072mm3
Tumor volume: 1013mm3
M10
M9
M11
M8
Figure S3: Individual time intensity curves (TIC) of the PC3pip orthotopic tumor after i.v. administration of PSMA-targeted NBs and non-targeted NBs.

## Slide 5
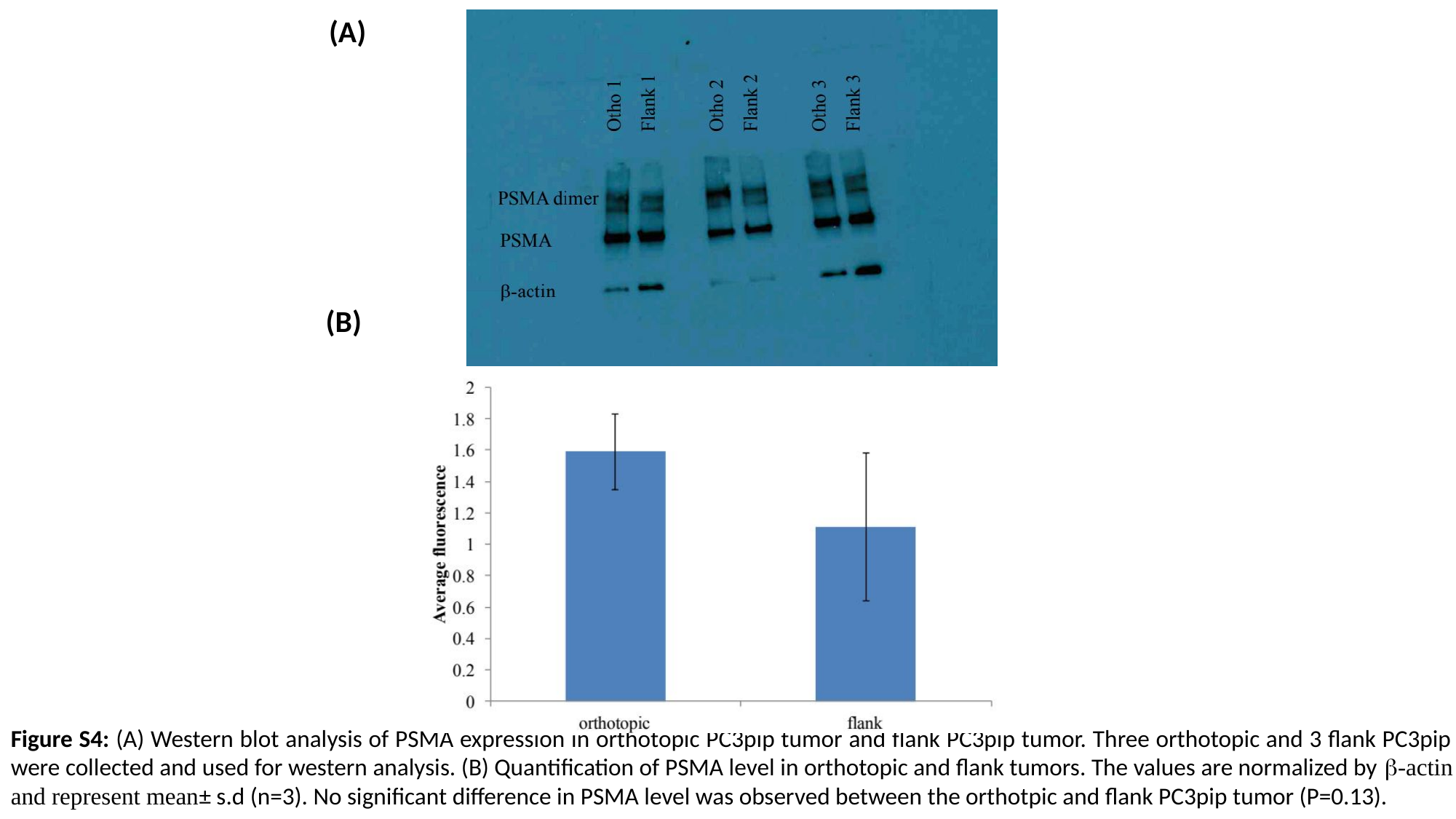

(A)
(B)
Figure S4: (A) Western blot analysis of PSMA expression in orthotopic PC3pip tumor and flank PC3pip tumor. Three orthotopic and 3 flank PC3pip were collected and used for western analysis. (B) Quantification of PSMA level in orthotopic and flank tumors. The values are normalized by β-actin and represent mean± s.d (n=3). No significant difference in PSMA level was observed between the orthotpic and flank PC3pip tumor (P=0.13).

## Slide 6
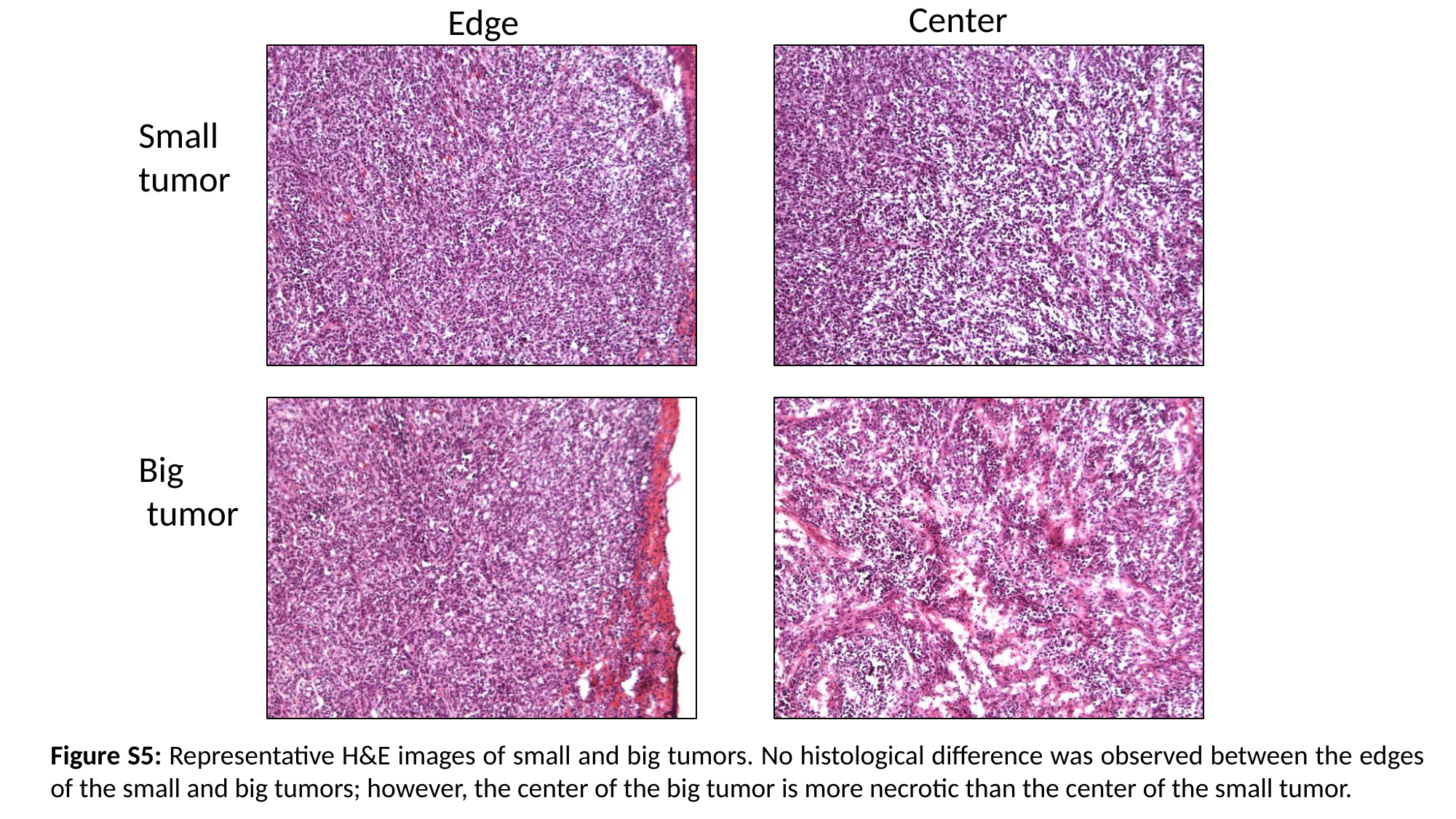

Center
Edge
Small tumor
Big
 tumor
Figure S5: Representative H&E images of small and big tumors. No histological difference was observed between the edges of the small and big tumors; however, the center of the big tumor is more necrotic than the center of the small tumor.
